# Viral and Host Factors Required for Avian H5N1 Influenza A Virus Replication in Mammalian Cells

**DOI:** 10.3390/v5061431

**Published:** 2013-06-10

**Authors:** Hong Zhang, Benjamin G. Hale, Ke Xu, Bing Sun

**Affiliations:** 1 Molecular Virus Unit, Key Laboratory of Molecular Virology and Immunology, Institut Pasteur of Shanghai, Shanghai institutes for Biological Sciences, Chinese Academy of Sciences, 225 South Chongqing Road, Shanghai 200025, China; E-Mail: zhanghong@sibs.ac.cn; 2 Medical Research Council (MRC), University of Glasgow Centre for Virus Research, 8 Church Street, Glasgow, G11 5JR, Scotland, UK; E-Mail: ben.hale@glasgow.ac.uk; 3 Laboratory of Molecular Cell Biology, Institute of Biochemistry and Cell Biology, Shanghai Institutes for Biological Sciences, Chinese Academy of Sciences, 320 Yueyang Road, Shanghai 200031, China

**Keywords:** avian H5N1influenza A viruses, replication in mammalian cells, molecular mechanisms, adaptive mutation, species barrier

## Abstract

Following the initial and sporadic emergence into humans of highly pathogenic avian H5N1 influenza A viruses in Hong Kong in 1997, we have come to realize the potential for avian influenza A viruses to be transmitted directly from birds to humans. Understanding the basic viral and cellular mechanisms that contribute to infection of mammalian species with avian influenza viruses is essential for developing prevention and control measures against possible future human pandemics. Multiple physical and functional cellular barriers can restrict influenza A virus infection in a new host species, including the cell membrane, the nuclear envelope, the nuclear environment, and innate antiviral responses. In this review, we summarize current knowledge on viral and host factors required for avian H5N1 influenza A viruses to successfully establish infections in mammalian cells. We focus on the molecular mechanisms underpinning mammalian host restrictions, as well as the adaptive mutations that are necessary for an avian influenza virus to overcome them. It is likely that many more viral and host determinants remain to be discovered, and future research in this area should provide novel and translational insights into the biology of influenza virus-host interactions.

## 1. Introduction

Influenza viruses are enveloped viruses containing a segmented negative-sense RNA genome, and belong to the *Orthomyxoviridae* family [1]. They can be classified into three types: A, B, and C [2,3], among which influenza A viruses have the most genetic variation and the broadest host range. Influenza A viruses are further divided into subtypes by the hemagglutinin (HA) and neuraminidase (NA) genes they possess. To date, 16 types of HA and nine types of NA have been identified in viruses from wild birds, while a putative 17th HA was recently discovered in fruit bats [4]. Meanwhile, more than 100 possible HA-NA combinations (HnNn; where n represents a particular subtype, e.g., H5) have been found in nature [5]. As influenza A viruses are long-established and frequently asymptomatic pathogens in wild birds, it is believed that aquatic birds serve as the natural reservoir for influenza A viruses. Phylogenetic analyses of viral genes further indicate that all current mammalian influenza A viruses are ultimately derived from avian influenza viruses [3]. In contrast to the vast gene pool in avian influenza A viruses, only a few different HnNn subtypes have been isolated from non‑avian hosts (e.g., humans, pigs, horses) suggesting that influenza A viruses are more ‘transient’ pathogens in mammalian hosts, and that significant species adaptation is needed [3]. For instance, only H1N1, H2N2, and H3N2 have successfully adapted to, and circulated, in humans during the past 100 years [6]. Considering the existence of a large avian influenza virus gene pool, and the frequent interspecies transmission of viruses between birds and mammalian species, it is possible that more subtypes will establish themselves as future mammalian influenza viruses. Indeed, some typically avian influenza viruses (e.g., H7N7 [7], H7N3 [8,9], H9N2 [10], and H5N1 [11]) have been reported to infect and cause disease in individual human cases, although human-to-human transmission was limited (if any), and certainly not sustained. The apparently high case-fatality rate of highly‑pathogenic H5N1 influenza virus in humans (50%–60% by WHO criteria [12]) has led many to fear that evolution of a mammalian-transmissible H5N1 virus will cause a potentially severe human pandemic.

The influenza A virus genome consists of eight RNA segments, and encodes 10 well-described proteins [2]. Furthermore, there are a growing number of newly identified proteins encoded by certain strains of influenza A virus, including PB1-F2 [13], PB1-N40 [14], PA-X [15], N-truncated PAs [16], M42 [17], and NS3 [18], which are generated by various co-transcriptional or co-translational strategies. The 10 common viral proteins are expressed in all influenza A viruses, and are functionally essential for a complete infection cycle in immuno-competent hosts. The HA protein present on the surface of the virion recognizes, and binds to, sialic acid on the surface of host cells, facilitating entry of the virus [5]. After binding, virus particles are endocytosed, and the lowering of the pH, caused by subsequent endosome maturation, triggers a conformational change in HA resulting in fusion of the endosome and virion membranes. The viral M2 protein (matrix 2) functions as an ion channel to further lower the pH of the virus particle, thereby aiding in the dissociation of the M1 protein (matrix 1) virion ‘shell’ such that the eight vRNPs (NP [nucleoprotein]-coated and polymerase complex [PB1, PA, and PB2]-bound viral RNAs) are released into the cytosol [19]. The vRNPs are then transported into the cell nucleus where accessory cellular components essential for influenza viral replication and transcription are located. After genome replication, transcription, and protein synthesis, NEP (nuclear export protein), and M1, act to traffic newly synthesized vRNPs out of the nucleus, into the cytoplasm, and to the plasma membrane, where assembly of progeny virions occurs. At these sites, several viral proteins contribute to budding, including M1 and M2. Finally, NA acts to remove sialic acid from glycoproteins in both the viral and cell membranes, thereby preventing interaction between HA and host cell receptors, and thus, ensuring release of new infectious virus particles [1]. NS1 (nonstructural protein 1) acts within the infected cell to counteract innate host-cell defense systems, including interferon (IFN), that may otherwise limit efficient virus replication [20].

When an interspecies transmission event occurs, the virus population will face a new host environment and selection pressure. In this scenario, only strains harboring mutations that are beneficial in the new species are likely to propagate efficiently and transmit within the new host population. These mutations will include those that ensure optimized viral functions for producing progeny virus and for disabling immune defenses in the new host. Although avian H5N1 influenza viruses are not entirely adapted to replicate and transmit in humans yet, infection of individual humans has occurred, sometimes with severe consequences. In this review, we summarize known viral and host-cell factors implicated in allowing avian H5N1 influenza viruses to establish successful infections in mammalian cells, and briefly discuss adaptive mutations in the viral genome that may be necessary to allow this to occur.

## 2. Molecular Mechanisms of Avian H5N1 Influenza A Virus Replication in Mammalian Cells

### 2.1. Crossing the Plasma Membrane

The plasma membrane is the first cellular barrier that protects a host cell from virus infection. Influenza A viruses must recognize specific receptors (sialic acids) in order to begin the infection of a cell [21]. There are two major types of linkages between sialyloligosaccharides (SAs) and galactose (Gal): SA-α2, 6-Gal and SA-α2, 3-Gal [21]. Typically, the HA proteins of human influenza viruses preferentially bind the SA-α2, 6-Gal linkage, while avian influenza virus HA proteins have a higher affinity for SA-α2, 3-Gal [22]. Thus, the tissue distribution of different sialic acid linkages is a major factor in determining the sites of initial virus infection and replication. SA-α2, 3-Gal is abundantly expressed on avian intestinal and respiratory epithelial cells, but is only expressed in the lower, and not upper, respiratory tracts of humans [23]. SA-α2, 6-Gal is mainly distributed on human airway epithelial cells of the upper respiratory tract, but can be detected in the respiratory and intestinal tracts of several avian species [21,22,23]. Typical avian influenza viruses, including H5N1, recognize SA-α2, 3‑Gal as their receptors and efficiently target and replicate in the intestinal tracts of birds, but not in the upper respiratory tracts of humans. Nevertheless, efficient virus replication in the human upper respiratory tract would be more likely to result in aerosol-mediated human-to-human transmission. In this regard, the avian-origin HA proteins from the 1918, 1957, and 1968 human pandemic strains all recognized the SA-α2, 6-Gal receptors [24,25]. This may suggest that these HA proteins had switched their sialic-acid binding specificity at some point during their adaptation to humans [24]. Thus, it is widely believed that avian H5N1 viruses would also have to change their receptor binding preference from SA-α2, 3-Gal to SA-α2, 6-Gal in order to infect humans and transmit efficiently. Current human infections with H5N1 viruses that target SA-α2, 3-Gal may lead to virus replication in the lower respiratory tracts of humans, thereby causing severe lung damage and disease. It cannot be excluded that such SA-α2, 3-Gal-targeted viruses could also transmit between humans, but as discussed below, this would require novel routes of transmission not commonly associated with human influenza.

A leucine residue at position 226 of the HA receptor binding domain is necessary for recognition of the SA-α2, 6-Gal receptor, but glutamine at this site permits the recognition of SA-α2, 3-Gal [21,24,26,27]. Fortunately, HAs from naturally isolated H5N1 influenza viruses still retain the avian‑type residue that preferentially binds SA-α2, 3-Gal, suggesting a molecular explanation for lack of transmission of these viruses between humans. However, other single amino-acid mutations, such as N154S [28], N182K [29], Q192R [29], Q222L [28], S223N [28], G224S [28], S227N [30], G228S [31], and combinations of mutations [32] surrounding the receptor binding pocket, have been shown to increase the SA-α2, 6-Gal binding activity of recombinant H5N1 influenza viruses experimentally. Thus, it is possible that some H5N1 viruses could naturally acquire an altered receptor binding preference, which would be selected in a future adaptation process. Indeed, there is evidence that H5N1 viruses are already delicately placed to undergo an adaptive process in chickens. Compared with avian viruses from aquatic birds, H5N1 viruses isolated from domestic chickens, and a fatal human case, showed low affinities towards both chicken red blood cells and soluble sialylglycoproteins, which partially resulted from additional glycosylation sites on the head of HA [33]. Thus, chickens are proposed to be a potential intermediate host for introduction of viruses from wild aquatic birds to humans [33]. Instead of completely switching HA binding affinity from SA‑α2, 3‑Gal to SA-α2, 6-Gal, H5N1viruses could still pose a replication/transmission threat in mammals by using alternative transmission routes where the initial infection is mediated bySA-α2, 3‑Gal receptors. It has been suggested that both respiratory and oral routes of transmission could contribute to the initial H5N1 avian-to-mammal cross-species jump [32]. In the respiratory route of transmission, not only can respiratory epithelial cells serve as susceptible cells, but subsequent neuronal transmission from the nasal cavity to olfactory bulb is a possibility [34]. In the oral route of transmission, the digestive and intestinal tracts of mammals may support some H5N1 virus replication [32,35]. Neurons harboring SA-α2, 3-Gal receptors on their surfaces are abundant in the wall of mammalian intestinal tract, and may permit the initial binding of avian H5N1 viruses if present and viable after ingestion. However, there is no evidence that avian influenza viruses commonly utilize the human intestinal tract as the initial site of infection. Nevertheless, mammalian cells harboring SA‑α2, 3-Gal receptors have the potential to represent a cell population that allows the initial attachment and replication of H5N1 viruses with SA‑α2, 3-Gal binding affinities.

### 2.2. Nuclear Import of NP (Nucleoprotein)-Coated and Polymerase Complex (PB1, PA, and PB2)-Bound Viral RNAs (vRNPs)

There are two ways for proteins to be transported into the cell nucleus: passive diffusion (for small proteins of ≤60–70 kDa) and energy-dependent import (for larger proteins and complexes, such as the influenza virus vRNP) [36]. The latter process occurs in a highly selective manner, dependent on the recognition of NLSs (nuclear localization signals) in imported cargoes by the host factors importin-α and importin-β. Usually, importin-β requires importin-α to bind directly to the NLS of the cargo proteins, thus serving as an adaptor, before the entire importing complex is transported into the cell nucleus [37]. In some cases, importin-β is also able to bind directly to cargoes independently of importin-α [38]. 

As transcription and replication of the influenza viral genome occurs in the nucleus, both viral RNAs and viral proteins have to be imported into the cell nucleus. After uncoating, the incoming vRNPs are imported into the nucleus as complexes, a process likely mediated by direct binding of NP to importin-α [39]. Subsequent newly synthesized viral polymerase and NP proteins are imported separately into the nucleus to assemble into new vRNPs during replication. NP contains two types of NLS: the unconventional NLS1 (aa1-13) and the bipartite NLS2 (aa198-216) [40,41]. The nuclear import of NP is mainly mediated by binding of importin-α to NP NLS1, which is more exposed than NLS2 [39,42]. The nuclear translocation of PB2 also depends on an interaction with importin-α [43,44]. In contrast, the nuclear import of monomeric PB1 and PB1-PA dimers relies on their interaction with the importin-β homologue RanBP5, which is independent of importin-α binding [45]. Notably, interactions between viral proteins are also important for the nuclear accumulation of some viral proteins. For example, an NLS in the N-terminal domain of PA is important for the nuclear accumulation of PB1 [46]. 

Although it might be expected that the nuclear import machinery is highly conserved between species given its fundamental nature, it appears that functional differences do exist. Therefore, influenza viruses must adapt this aspect of their replication when establishing themselves in a new host. An excellent example of how an avian influenza virus can accumulate adaptive mutations to allow it to cross the mammalian nuclear envelope is illustrated by experiments using the mouse-adapted virus SC35M, and its avian ancestor strain SC35 (H7N7) [47]. Adaptive mutations in both PB2 (D701N) and NP (N319K) make the SC35M strain better adapted to the mammalian nuclear import system, as the mutant viral proteins bind more tightly to importin-α1 and -α7 in mammalian cells than the parental viral proteins of SC35. This enhanced binding is paralleled by increased transport of PB2 and NP into the nucleus of mammalian cells [47,48]. It has been proposed that other mutations in PB2 may also increase its binding to importins, but whether the enhanced interaction up-regulates nuclear import needs further clarification [49]. 

### 2.3. Replication in the Nucleus

After entry into the nucleus, transcription and replication of the influenza virus genome depends on viral polymerase activity, together with cellular co-factors. Since the outbreak of highly‑pathogenic H5N1 avian influenza virus in 1997, avian-type viruses containing the PB2 E627K mutation have been increasingly isolated from human H5N1 infection cases, and this mutation has been identified as a mammalian adaptive marker [50]. The underlying mechanism of mammalian adaptation by PB2 E627K is still unclear, although multiple possibilities have been suggested. It has been hypothesized that ‘mammalian-type’ PB2 627K is beneficial for the replication of avian influenza viruses in the human upper respiratory tract, which generally has a temperature of only 33 °C. In contrast, the avian intestinal tract is closer to 41 °C, a temperature at which the ‘avian-like’ PB2 627E polymorphism facilitates efficient virus replication [51,52]. Additionally, NP appears to bind more efficiently to PB2 627K than 627E in mammalian cells, but not in avian cells [53], which is likely responsible for enhancing polymerase activity. 

The E627K substitution is not the only mammalian-adaptive mutation that can arise in the viral polymerase. Indeed, a large proportion of H5N1 strains isolated from humans possess PB2 627E, suggesting that other adaptive amino-acid substitutions must exist in order to counterbalance its generally low polymerase activity in human cells. It is reported that PB2 591K/R and PB2 701N can compensate for lack of PB2 627K, thereby ensuring efficient polymerase activity in mammalian cells [47,54]. There is also evidence that the PB1 gene from an avian source promotes avian polymerase activity in mammalian cells [55]. Two mutations in PB1 (473V and 598P) were identified as increasing the polymerase activity of viruses carrying PB2 627E in mammalian cells [56]. More recently, it was found that mutations in NEP are also involved in host adaptation. The adaptive mutation M16I (and others) in the NEP proteins of certain human H5N1 isolates can increase the relatively low polymerase activity of avian viruses in mammalian cells [57]. These NEP mutations are more common in human H5N1 isolates carrying the PB2 627E mutation than in human H5N1 viruses possessing PB2 627K, which suggests that NEP can act as an important determinant of host adaptation by promoting efficient polymerase activity in human cells. 

From the host perspective, it is interesting to note that cellular factors that differentially regulate influenza A virus polymerase activity depending on the PB2 627K/E status have been identified by RNAi [58]. Among these factors, DDX17, a member of the DEAD-box RNA helicase family, was found to promote general viral replication in a host dependent manner. While avian DDX17 appears to be essential for efficient avian influenza virus polymerase activity, human DDX17 inhibits avian polymerase activity. In human cells, human DDX17 can only support the polymerase activities of H5N1 polymerase complexes carrying the PB2 627K, but not 627E, mutation. Thus, optimal replication of avian influenza viruses in mammalian cells may depend on DDX17 and its functional interaction with PB2.

Another important host factor is importin-α, which has been discussed above for its nuclear transport functions. Beyond simply importing viral proteins into the cell nucleus, importin-α also contributes to host-specific optimal virus replication in an isoform dependent manner [48]. Efficient replication of the avian SC35 virus (with avian signature residues PB2 701D and NP 319N) depends on importin-α1 and -α3, while replication of the mammalian-adapted SC35M virus (with mammalian signature residues PB2 701N and NP 319K) depends on importin-α1 and α7 [48]. With regards to PB2 627 polymorphisms, it was found that polymerase activity of viruses containing PB2 627K is enhanced by recruitment of mammalian importin-α1 and importin-α7, although subcellular localization of PB2 is not affected [59]. Notably, it has been demonstrated that the replication of an H5N1 virus, isolated from a lethal human case (A/Thai/KAN-1/04), relies on importin‑α7 but not importin-α3 in human cell culture models, even though the PB2 of this virus binds importin-α7 poorly [48]. These studies suggest that a switch from importin-α3 to importin-α7 specificity occurs during efficient avian-to-mammal adaptation, and that viruses may utilize multiple functions of importin-α isoforms, not just its well-characterized nuclear import activity [49].

A limited number of studies have identified distinct mammalian restriction factors for the influenza viral polymerase that appear to act independently of PB2 627K. Human nuclear protein 90 (NF90), a dsRNA-binding protein, can co-purify with the viral NP protein, an interaction that negatively regulates both avian and human influenza A virus replication and polymerase activities in human cells [60]. In contrast, the human and murine interferon-inducible Mx proteins (a dynamin-like large GTPase), seems to specifically limit avian influenza virus replication [61]. The increased sensitivity of avian H5N1 viruses to the antiviral effects of human and murine Mx proteins, as compared with human influenza viruses, has been mapped to the NP protein, suggesting that specific adaptive mutations in NP have to be acquired for avian influenza viruses to overcome an important mammalian innate immune restriction [62]. 

Taken together, to replicate efficiently in the mammalian nucleus, avian influenza A viruses must gain adaptive mutations for elevated polymerase activity, and/or mutations to support the recruitment of positively acting host factors (e.g., DDX17 and importin-α7). In addition, avian viruses may only succeed in a mammalian host if they adapt to overcome specific host restriction factors (e.g., Mx). It is evident that currently circulating H5N1 strains possess only some of these capabilities, thereby allowing them to overcome only a few described host barriers. Further studies to map adaptive mutations in fine detail, with respect to such barriers, will provide the essential tools to maintain global surveillance for emerging H5N1 viruses.

### 2.4. Escaping the Host Immune Response

During infection, the host innate immune response is elicited to counteract virus replication. Influenza A viruses limit this cellular defense mechanism in infected cells by producing the NS1 protein from genomic segment 8. NS1 is highly multifunctional, but is well-characterized as an antagonist of the host antiviral response, especially by repressing the production of interferons (IFNs) [20]. NS1 acts in several ways to limit IFN production during infection. Firstly, NS1 can disrupt the cytoplasmic RIG-I-mediated signaling pathway that would otherwise lead to primary transcription of IFN mRNA. Simultaneously, NS1 protein molecules in the nucleus can inhibit the post‑transcriptional maturation of IFN mRNA by interacting with the host-cell pre-mRNA cleavage and polyadenylation specificity factor, CPSF30 [20]. 

In recent years, it has become increasingly apparent that a number of NS1 functions are virus strain‑specific. All NS1 proteins appear to bind and neutralize RIG-I ligands (such as 5′‑triphosphated RNAs) as part of their antagonism of RIG-I signaling [63]. However, another mechanism by which NS1 proteins can inhibit RIG-I activation is by preventing the ubiquitination of RIG-I by either TRIM25 or Riplet ubiquitin E3 ligases, and this function appears to vary between strains [64]. Studies suggest that an avian-type H5N1 NS1, but not human/swine-type NS1s, can bind efficiently to chicken TRIM25 to suppress IFN production in chicken cells. In contrast, all NS1s tested so far (including avian, swine, and human) appear to interact with human TRIM25. Interestingly, the avian H5N1 NS1 protein used in the above study failed to interact with human Riplet, while human‑type NS1 proteins bind human Riplet and inhibit its RIG-I activating function [64]. It remains to be determined precisely whether the various different NS1-TRIM25/Riplet binding specificities contribute to avian-mammalian transmission.

The interaction of NS1 with CPSF30 is also strain-dependent. With respect to avian H5N1 viruses that have sporadically entered the human population, the NS1 proteins from early viruses isolated around 1997 possessed an intrinsic defect in binding human CPSF30. However, since 1998, mutations selected for at NS1 residues 103 and 106 have lead to H5N1 viruses acquiring the ability to bind human CPSF30 and, thereby, better suppress the human IFN response [65]. The exact selection pressure causing this phenomenon is unclear, but acquisition of such a function can increase the virulence of avian H5N1 viruses in mammalian hosts [66].

Sequence analyses have revealed several features of NS1 that are potentially relevant to host adaptation. Firstly, the NS1 gene can be divided into two major alleles: A and B [20]. Allele A NS1s are found in both avian and human viruses, while allele B NS1s are only found in avian viruses [67,68]. Viruses with allele A NS1s appear to have a replication advantage over those with allele B NS1s in mammalian cells, suggesting that those avian H5N1 influenza viruses carrying allele A NS1 genes may already have acquired functions adapted to work in mammals. A second key NS1 feature revealed by sequencing is the presence of strong or weak PDZ-ligand domain consensus motifs located at the C‑terminus of NS1. Such motifs have been identified as virulence determinants [69]. By interacting with host PDZ-containing proteins, certain NS1s can interfere and disrupt multiple host signaling pathways, including IFN-stimulated signaling, tight junctions, and apoptosis [69,70,71,72]. Generally, NS1 proteins with avian-like PDZ-ligand domains (including H5N1) are associated with high virulence in mammals [70], which may result from binding of these ‘strong’ PDZ-ligand domains to several human PDZ-containing proteins [73]. Therefore, it is possible that switching from avian‑type (‘strong’) to human-type (‘weak’) PDZ-ligand domain motifs is required during full avian‑to-mammal adaptation as a virus reaches equilibrium with its host and virulence is compromised. The C-terminus of NS1 is thought to be unstructured [74], and is highly variable in length between strains [68,75]. Different variants may have different host-specific preferences relating to cellular binding partners, which will include cellular PDZ-domain containing proteins as well as other proteins. Whether the C-terminal length variability of NS1 proteins contributes to host adaptation remains to be thoroughly examined.

### 2.5. Release from the Cell Surface

The viral NA protein removes sialic acid from the surface of host cells, from which virus particles are budding. This minimizes any HA-mediated linkage between the virion and host cell receptors, thus enabling efficient virus release from the cell surface. NA also removes sialic acid from glycoproteins embedded within the viral membrane in order to prevent HA-mediated aggregation of virions. The NA proteins from human and avian influenza viruses can have different sialic-acid substrate specificities, which often correlate with the receptor binding specificities and affinities of their associated HAs [76]. The NAs from avian viruses have relatively higher enzymatic activity to cleave SA-α2, 3-Gal linkages, whereas human influenza virus NAs can more efficiently cut SA-α2, 6-Gal linkages. Notably, the avian-derived NA from human H2N2 virus, in which HA recognized SA-α2, 6-Gal, gradually drifted from hydrolyzing SA-α2, 3-Gal linkages to SA-α2, 6-Gal linkages during the period it was circulating in humans after 1957 [77,78]. Indeed, an alignment of HA and NA specificities exists in influenza viruses that are well adapted to a particular host [76]. 

In addition to HA and NA substrate specificity alignment, the receptor binding strength of HA needs to be balanced with the enzymatic activity of NA. Genome sequencing of H5N1 viruses isolated from poultry farms, after outbreaks of human H5N1 virus infections, has shown that H5N1 viruses containing a short-stalked NA accompanies an HA glycosylated at position 158 [33,79]. Although the short‑stalked NAs exhibit low enzymatic activity due to inefficient positioning of the substrate in the active site [80], the accompanying glycosylation on HA residue 158 also reduces the affinity between HA and host receptors. This type of combination seems to be beneficial for avian influenza viruses to replicate efficiently in chicken cells [79], and H5N1 viruses with short-talked NAs are more virulent in mice than viruses with longer-stalk NAs [81]. Thus, the adaptation of avian influenza viruses to a new host requires simultaneous functional changes in HA and NA to achieve balanced receptor binding and cleavage activity. 

NA proteins can be related to the pathogenicity of certain avian H5N1 influenza viruses by other mechanisms. Transforming growth factor-β1 (TGF-β), a multifunctional molecule, regulates the host immune response [82]. TGF-β is secreted as an inactive form known as latent TGF-β (LTGF-β), which must be activated to form functional TGF-β. The level of active TGF-β has been reported to increase during influenza A virus infection due to NA cleaving LTGF-β to release active TGF-β. However, the NAs from certain highly pathogenic H5N1 avian influenza viruses lack the ability to activate TGF-β *in vivo* and *in vitro* without affecting other NA functions [82]. Thus, it seems that avian and human NAs can differ in their activities relating to both virus particle release and immune response regulation, which may have important implications for cross-species transmission and virulence.

## 3. Summary

Herein, we have summarized current knowledge pertaining to molecular mechanisms involved in efficient mammalian infection by avian H5N1 influenza A viruses. We have focused on both the physical and functional barriers that such viruses must cross when entering a new host, including the mammalian cell membrane, the nuclear envelope, the maintenance of polymerase activity in mammalian nuclei, the host innate immune response, and the release from mammalian cells. To completely overcome these restrictions, an avian H5N1 influenza virus must acquire several adaptive mutations in multiple segments of its genome (see Table 1). Although the currently circulating avian H5N1 strains are not yet fully adapted to humans, several partial adaptations appear to have already arisen, although the selection pressures acting to drive such changes (if any) are unknown. Nevertheless, given the infidelity of RNA viruses such as influenza virus, natural acquisition of further adaptive mutations while it is circulating in its natural avian hosts is highly possible. Recent ground‑breaking studies have shown that only very few adaptive mutations in an avian H5N1 influenza virus are required in order to make it airborne-transmissible in mammalian models, confirming the pandemic potential of avian H5N1 viruses [83,84]. The adaptive changes identified in these experimental studies included mutations in the HA and PB2 proteins, underlining the species-specific functional importance of polymorphisms in these two viral proteins. Future work to dissect the fundamental molecular mechanisms required for efficient H5N1 avian influenza A virus replication in mammalian cells will help to identify key virulence or adaptation markers that can be used during global surveillance of viruses threatening to emerge into the human population. In addition, such studies may provide insights into new opportunities to combat such a cross-species transfer.

**Table 1:** Amino acid mutations involved in mammalian infection with H5N1 virus.

Viral components	Protein	Mutation	Adaptive Mechanism in Mammalian System	Reference
Surface glycosylation protein	HA	N154S	Increase α2, 6 binding of H5N1 subtype	[28]
		A158T	Decrease α2, 3 binding by oligosaccharide modification of H5N1 subtype	[33]
		N182K	Increase α2, 6 binding and decrease α2, 3 binding of H5N1 subtype	[29]
		Q192R	Increase α2, 6 binding and decrease α2, 3 binding of H5N1 subtype	[29]
		Q222L	Increase α2, 6 binding and decrease α2, 3 binding of H5N1 subtype	[28]
		S223N	Increase α2, 6 binding and decrease α2, 3 binding of H5N1 subtype	[28]
		G224S	Increase α2, 6 binding and decrease α2, 3 binding of H5N1 subtype	[28]
		Q226L	Increase α2, 6 binding and decrease α2, 3 binding of H2,H3,H5,H9 subtype	[21,24,26,27]
		S227N	Increase α2, 6 binding and decrease α2, 3 binding of H5N1 subtype	[30]
		G228S	Increase α2, 6 binding of H5N1 subtype	[31]
	NA	Deletion in stalk	Functional balance with HA by decreasing enzyme activity of H5N1 subtype	[33,79]
Polymerase	PB1	L473V	Increase polymerase activity of H5N1 and 2009pH1N1 subtype	[56]
		L598P	Increase polymerase activity of H5N1 subtype	[56]
	PB2	Q591K	Increase polymerase activity of H5N1 and 2009 pH1N1 subtype	[54]
		E627K	Increase polymerase activity of H5N1 subtype	[51,52]
		D701N	Increase polymerase activity by binding to beneficial importin α isoform in H5N1 and H7N7 subtype	[47]
Non-structural protein	NS1	PDZ domain in C-terminus	Bind to host PDZ-carrying proteins to interfere host signal pathway	[69,70,71,72]
		S103F, I106M	Bind to host CPSF30 to inhibit protein synthesis	[65,66]
Nuclear export protein	NEP	M16I	Increase polymerase activity of H5N1 subtype	[57]
